# Pathological Features of Diabetic Retinopathy in Spontaneously Diabetic Torii Fatty Rats

**DOI:** 10.1155/2019/8724818

**Published:** 2019-09-15

**Authors:** Yoshiaki Tanaka, Rina Takagi, Takeshi Ohta, Tomohiko Sasase, Mina Kobayashi, Fumihiko Toyoda, Machiko Shimmura, Nozomi Kinoshita, Hiroko Takano, Akihiro Kakehashi

**Affiliations:** ^1^Department of Ophthalmology, Jichi Medical University, Saitama Medical Center, 1-847 Amanuma-cho, Omiya-ku, Saitama, Saitama 330-8503, Japan; ^2^Biological/Pharmacological Research Laboratories, Central Pharmaceutical Research Institute, Japan Tobacco Inc., 1-1 Murasaki-cho, Takatsuki, Osaka 569-1125, Japan; ^3^Toyoda Eye Clinic, 7-1-10, Kisicho, Urawa-ku, Saitama, Saitama, Japan

## Abstract

**Objective:**

The Spontaneously Diabetic Torii (SDT) fatty rat, established by introducing the *fa* allele (obesity gene) of the Zucker fatty rat into the SDT rat genome, is a new model of obese type 2 diabetes. We studied the pathologic features of diabetic retinopathy (DR) in this animal.

**Methods:**

The eyes of SDT fatty, SDT (controls), and Sprague Dawley (SD) rats (normal controls) were enucleated at 8, 16, 24, 32, and 40 weeks of age (*n* = 5‐6 for each rat type at each age). The retinal thicknesses, numbers of retinal folds, and choroidal thicknesses were evaluated. Immunostaining for glial fibrillary acidic protein (GFAP) and vascular endothelial growth factor (VEGF) was performed. Quantitative analyses of the immunopositive regions were performed using a cell-counting algorithm.

**Results:**

The retinas tended to be thicker in the SDT fatty rats and SDT rats than in the SD rats; the choroids tended to be thicker in the SDT fatty rats than in the SD rats. The retinal folds in the SDT fatty rats developed earlier and were more severe than in the SDT rats. Quantitative analyses showed that the GFAP- and VEGF-positive regions in the retinas of the SDT fatty rats were significantly larger than those of the SDT rats.

**Conclusions:**

SDT fatty rats developed more severe DR earlier than the SDT rats. The SDT fatty rats might be useful as a type 2 diabetes animal model to study DR.

## 1. Introduction

Diabetes has reached nearly epidemic levels worldwide. Many patients with diabetes with long histories of morbidity have one or more diabetic complications, such as diabetic nephropathy, diabetic peripheral neuropathy, or diabetic retinopathy (DR), all of which impact the patients' quality of life.

Among the ocular complications associated with diabetes, DR is a leading cause of visual loss and blindness in adults in developed countries [1]. Researchers need to determine how DR develops and preventative measures using animal models of diabetes. To that end, an animal model of diabetes with ocular complications that mimic human DR should be established.

Many diabetic animal models have been reported [2]. The Goto-Kakizaki (GK) rat is a nonobese animal with mild type 2 diabetes [3, 4]. Although electroretinography (ERG) showed functional abnormalities of photoreceptors in GK rats [5], no significant differences were observed in the retinal arterial and venous diameters [6]. Énzsöly et al. reported that degenerative changes in the photoreceptors and pigment epithelium developed in streptozotocin-induced diabetic rats [7]. Using male Wistar and Sprague Dawley (SD) rats, those investigators found no significant differences in the retinal thicknesses between the normal and diabetic rats. Long-Evans Tokushima Lean rats have been used as a model of type 1 diabetes [8, 9]. Although pancreatic changes and genetic analysis were discussed in those studies, no ocular complications were mentioned. Yang et al. [10] reported that the retinas of Otsuka Long-Evans Tokushima Fatty rats, a well-known model of type 2 diabetes, were significantly thinner than normal Long-Evans Tokushima Otsuka rats, and that tendency was apparent in the retinal nerve fiber layer using spectral-domain optical coherence tomography (OCT). While the ocular findings in the diabetic animal models in those studies are important to the understanding of the diabetic ocular complications, the ocular changes in those models differ markedly from those in humans. In particular, no retinal thickening occurs in most diabetic animals, unlike in patients with diabetic macular edema (DME).

A spontaneously type 2 diabetic strain of the SD rat, the Spontaneously Diabetic Torii (SDT) rat, was established in 1997 [11]. Hyperglycemia, nephropathy, and peripheral neuropathy have been reported in this rat [12]. We reported the severe diabetic ocular complications in this model [13–16]. The retinas tended to be thicker in SDT rats than in SD rats [16]. Mature diabetic cataracts and proliferative DR, especially, resemble human diseases in SDT rats [11, 15] and appear only in this diabetic rat model. We also studied the effect of ranirestat, an aldose reductase inhibitor, on DR in the SDT rats [17].

However, the systemic features and DR in the SDT rat differ somewhat from those in humans. It takes a long time to develop DR in the SDT rat. It has been reported that severe DR is found in 80% of the SDT rat at 51 to 60 weeks of age [13]. For that reason, a more suitable experimental animal model of DR is needed.

The SDT fatty rat, established in 2004 by introducing the *fa* allele (obesity gene) of the Zucker fatty rat into the SDT rat genome, is a new model of obese type 2 diabetes. The prominent findings of hyperglycemia, overt obesity, hyperlipidemia, and diabetes-related complications (nephropathy, peripheral neuropathy, etc.) develop at a younger age in SDT fatty rats compared to SDT rats [18, 19]. It is noteworthy that nephropathy develops in SDT fatty rats at 8 weeks of age, which is much earlier than in SDT rats at 24 weeks of age [18]. The SDT fatty rat is presumed to be a suitable animal model to reproduce clinical diabetes cases that have multiple metabolic disorders. In the retina, the peak latencies of the oscillatory potentials in ERGs in SDT fatty rats are prolonged compared with the age-matched normal SD rats, demonstrating retinal dysfunction [18]. In our preliminary study, the SDT fatty rats exhibited increases in the vascular endothelial growth factor (VEGF) concentrations in the vitreous humor and both retinal vascular hyperpermeability and retinal thickening, and those findings were normalized by phlorizin [20]. In the current study, we evaluated quantitatively and chronologically the pathological features of DR that developed in SDT fatty rats.

## 2. Methods

### 2.1. Animals

The care and handling of animals were in accordance with the Association for Research in Vision and Ophthalmology Statement for the Use of Animals in Ophthalmic and Visual Research, the Guidelines for Animal Experimentation of Japan Tobacco Inc., and the Guidelines for Animal Experimentation of the Animal Care and Committee of Jichi Medical University, the last of which approved all experiments (study number, 17095-01). We used colonies of male SDT fatty rats (*n* = 30), SDT rats (*n* = 30), and normal SD rats (*n* = 25) purchased from CLEA Japan Inc. (Tokyo, Japan). All SDT fatty and SDT rats were confirmed to be diabetic based on a nonfasting blood glucose concentration exceeding 350 mg/dL. The SDT rats were diagnosed with diabetes by 8 to 16 weeks after birth. The SDT fatty rats were diagnosed with diabetes by 8 weeks after birth. All rats were fed standard rat chow (CRF-1, Oriental Yeast Inc., Tokyo, Japan). The eyes were enucleated in SDT fatty and SDT (control) rats at 8, 16, 24, 32, and 40 weeks of age (*n* = 6 for each rat type at each age). The eyes of age-matched male SD rats (normal controls) also were enucleated (*n* = 5 at each age).

### 2.2. Measurement of Body Weight, Blood Glucose, Blood Insulin, Blood Triglycerides, and Blood Total Cholesterol

Body weight and blood chemistry parameters, including glucose, insulin, triglycerides (TG), and total cholesterol (TC), were measured when each rat was sacrificed. Blood samples were collected from the tail vein of nonfasting rats. The serum glucose, TG, and TC levels were measured using commercial kits (Roche Diagnostics, Basel, Switzerland) and an automatic analyzer (Hitachi 7180, Hitachi High-Technologies Corp., Tokyo, Japan). The serum insulin was measured using a rat-insulin enzyme-linked immunosorbent assay kit (Morinaga Institute of Biological Science, Yokohama, Japan).

### 2.3. Ocular Histopathology

The procedures of the histopathological study were the same as we reported previously [17]. Under deep isoflurane anesthesia (isoflurane inhalation solution, Pfizer Inc., New York, NY, USA), the eyes were enucleated for conventional histopathologic studies and placed in a fixative (Super Fix KY-500, Kurabo Industries Ltd., Osaka, Japan). The fixed eyes were washed in 0.1% mol/L cacodylate buffer and embedded in paraffin. The paraffin block was cut into 4 *μ*m sections that were stained with hematoxylin and eosin (HE) for conventional histopathologic examination. The immunohistochemical procedures were based on the standard avidin-biotin horseradish peroxidase method using each antibody and performed with 3,3′diaminobenzidine substrate-chromogen. Glial fibrillary acidic protein (GFAP) mouse monoclonal antibody (Cell Signaling Technology Inc., Danvers, MA, USA) and rat VEGF antibody (R&D Systems Inc., Minneapolis, MN, USA) were used at a dilution of 1 : 100 as the primary antibody.

### 2.4. Measurement of Retinal Thickness, Retinal Folds, and Choroidal Thickness

To quantify the pathological features of the specimens, we used the BZ-X700 digital microscope system (Keyence Corp., Osaka, Japan). One high-resolution image of an entire specimen was created using the BZ-H3XD image stitching system (Keyence). After staining with HE, the retinal thicknesses, numbers of retinal folds, and choroidal thicknesses were evaluated in the images. The total retinal thickness was defined as the distance between the retinal internal limiting membrane and the photoreceptor layer (PL). The mean retinal and choroidal thicknesses were measured 500, 1,000, and 1,500 microns from the optic nerve disc. The numbers of retinal folds, defined as deformations from the outer nuclear layer (ONL) to the PL, were measured within 1,500 microns of the optic nerve disc.

### 2.5. Measurement of the Area of Immunostained GFAP and VEGF

Quantitative analyses of the GFAP- and VEGF-positive regions, which we referred to as the immunopositive regions, were performed using the Hybrid Cell Count Module/BZ-H3C software (Keyence). The entire specimen was marked with a magenta stain, and the immunopositive regions were marked with dark blue over them. The boundary was marked with light blue. The color coding of the immunopositive and immunonegative regions can be selected freely in this software. The ratio of the immunopositive areas to the entire specimen was calculated automatically in each specimen.

### 2.6. Statistical Analysis

The measurements of the parameters are expressed as the mean ± standard error. For statistical analysis, we used the Excel Tokei 2006 software (Social Survey Research Information Co. Ltd., Tokyo, Japan). The Mann–Whitney *U*-test and Scheffe's test were used for the numerical parameter test of nonnormal distribution. *p* < 0.05 was considered significant.

## 3. Results

### 3.1. Body Weight, Blood Glucose, Blood Insulin, TG, and TC

**Figure 1:**
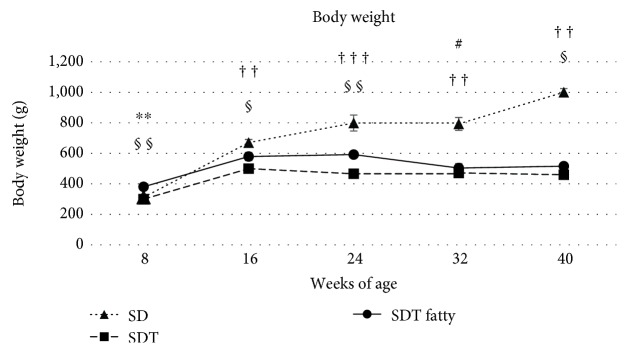
Body weight of the study animals. Compared with the Sprague Dawley (SD) rats, the Spontaneously Diabetic Torii (SDT) rats are significantly lighter. The SDT fatty rats are significantly heavier than the SDT rats. The data are expressed as the mean ± standard error. ^∗∗^*p* < 0.01, SDT fatty rats vs. SDT rats; ^#^*p* < 0.05, SDT fatty rats vs. SD rats; and ^††^*p* < 0.01 and ^†††^*p* < 0.001, SDT rats vs. SD rats by Scheffe's test. ^§^*p* < 0.05 and ^§§^*p* < 0.01, SDT fatty rats vs. SDT rats by Mann–Whitney *U*-test.

Figure 1 shows the changes in body weight. Compared with the SD rats, the SDT rats were significantly lighter (*p* < 0.01 at 16, 32, and 40 weeks of age; *p* < 0.001 at 24 weeks of age by Scheffe's test). The SDT fatty rats were significantly heavier than the SDT rats (*p* < 0.05 at 16 and 40 weeks of age; *p* < 0.01 at 8 and 24 weeks of age by Mann–Whitney *U*-test).

**Figure 2:**
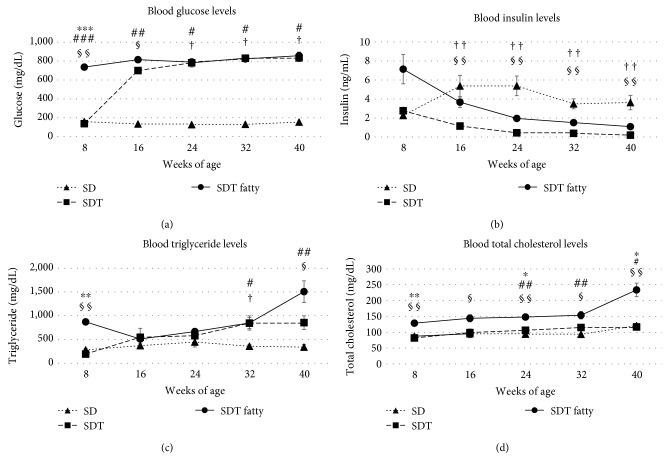
Changes in biologic parameters ((a) glucose, (b) insulin, (c) triglycerides, and (d) total cholesterol) in the three rat types. Glycolipid disorders in the Spontaneously Diabetic Torii (SDT) fatty rats are obviously prominent compared with those in the SDT rats. The data are expressed as the mean ± standard error. ^∗^*p* < 0.05, ^∗∗^*p* < 0.01, and ^∗∗∗^*p* < 0.001, SDT fatty rats vs. SDT rats; ^#^*p* < 0.05, ^##^*p* < 0.01, and ^###^*p* < 0.001, SDT fatty rats vs. Sprague Dawley (SD) rats; ^†^*p* < 0.05 and ^††^*p* < 0.01, SDT rats vs. SD rats by Scheffe's test. ^§^*p* < 0.05 and ^§§^*p* < 0.01, SDT fatty rats vs. SDT rats by Mann–Whitney *U*-test.

Figure 2 shows the changes in blood glucose, blood insulin, TG, and TC. The SDT rats were hyperglycemic from 16 weeks of age and the SDT fatty rats from 8 weeks of age. The mean insulin levels in the SDT fatty rats were higher than those in the SDT rats from 16 weeks of age. The mean serum TC levels in the SDT fatty rats were higher than those in the SDT rats at each age.

### 3.2. Retinal Thickness, Retinal Folds, and Choroidal Thickness

**Figure 3:**
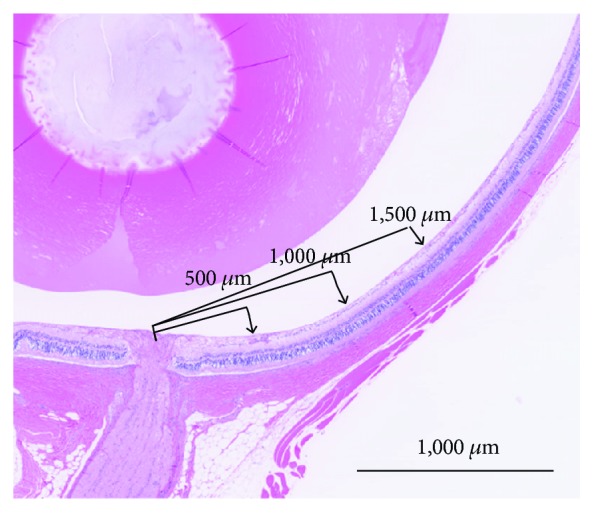
The retinal and choroidal thicknesses were measured 500, 1,000, and 1,500 microns from the optic nerve disc. The scale bar indicates 1,000 microns.

**Figure 4:**
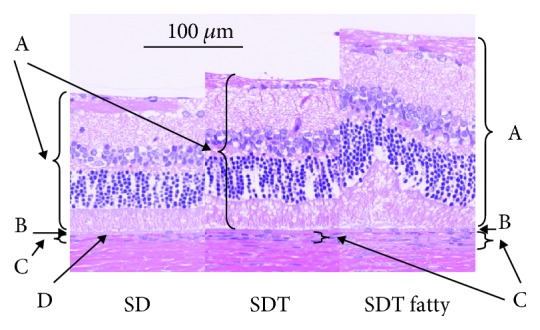
Comparison of the retinal and choroidal thicknesses 500 microns from the optic nerve disc at 40 weeks of age in each rat type (hematoxylin and eosin stain). A: retina; B: retinal pigment epithelium; C: choroid; D: choriocapillaris. The scale bar indicates 100 microns.

**Figure 5:**
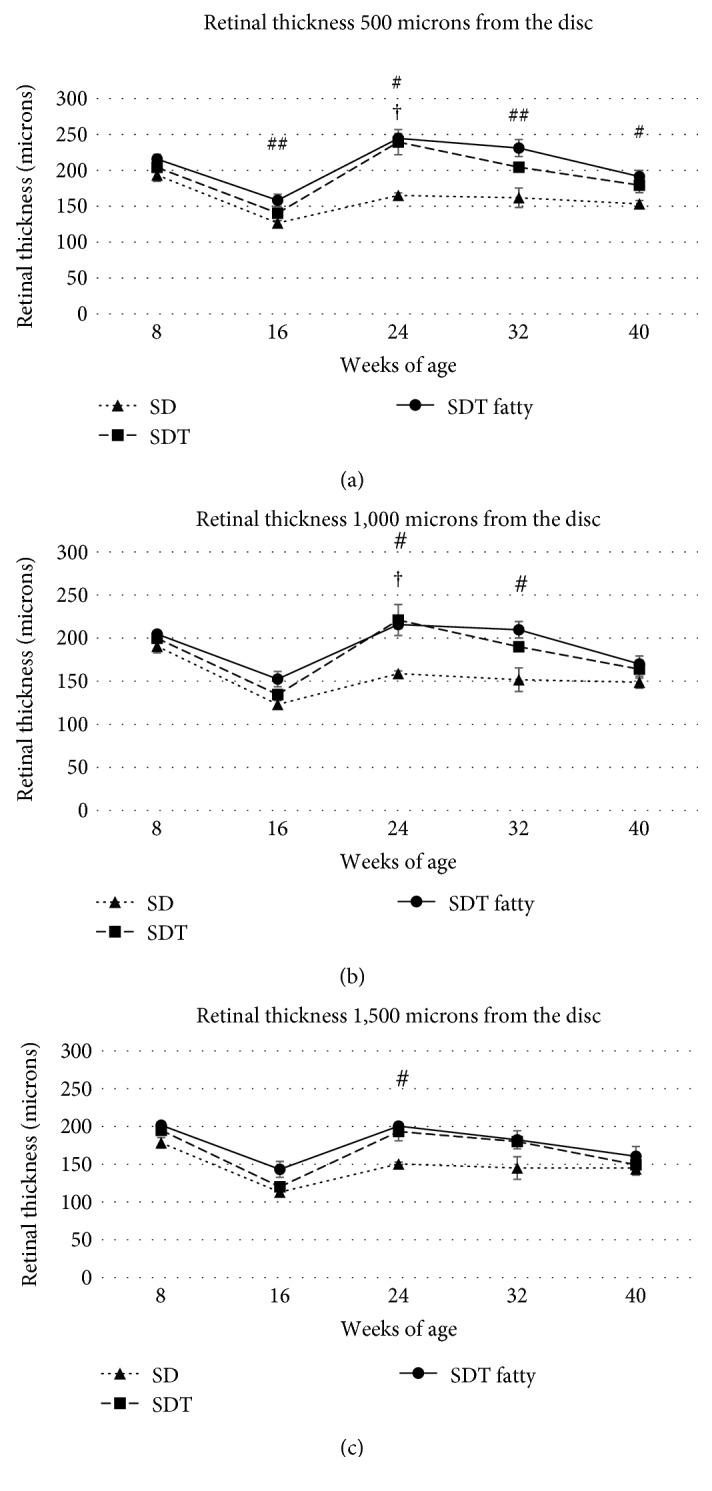
The retinal thicknesses ((a) 500 microns from the disc; (b) 1,000 microns from the disc; (c) 1,500 microns from the disc) in each rat type. The retinas tend to be thicker in the Spontaneously Diabetic Torii (SDT) fatty rats and SDT rats than in the Sprague Dawley (SD) rats. The data are expressed as the mean ± standard error. ^#^*p* < 0.05 and ^##^*p* < 0.01, SDT fatty rats vs. SD rats; ^†^*p* < 0.05, SDT rats vs. SD rats by Scheffe's test.

**Figure 6:**
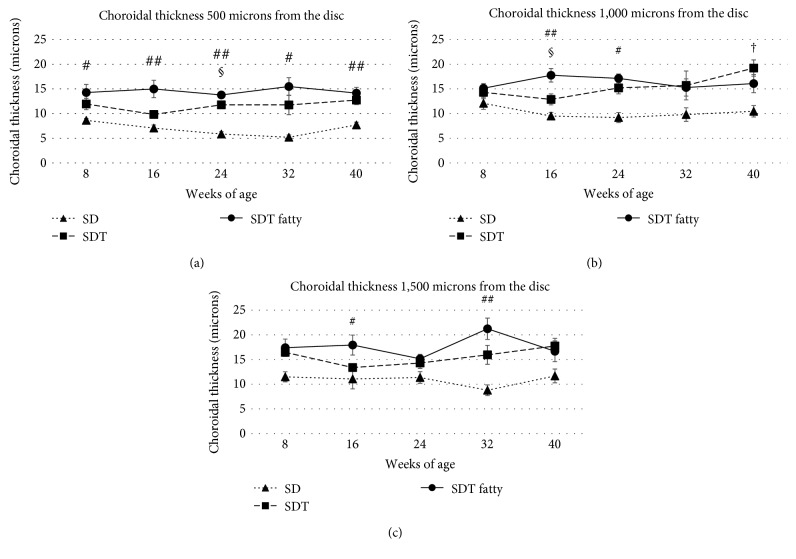
The choroidal thicknesses ((a) 500 microns from the disc; (b) 1,000 microns from the disc; (c) 1,500 microns from the disc) in each rat type. The choroids tended to be thicker in the Spontaneously Diabetic Torii (SDT) fatty rats than in the Sprague Dawley (SD) rats. The data are expressed as the mean ± standard error. ^#^*p* < 0.05 and ^##^*p* < 0.01, SDT fatty rats vs. SD rats; ^†^*p* < 0.05, SDT rats vs. SD rats by Scheffe's test. ^§^*p* < 0.05, SDT fatty rats vs. SDT rats by Mann–Whitney *U*-test.

Figures 3–6 show the retinal and choroidal thicknesses. The retinas tended to be thicker in the SDT fatty rats and SDT rats than in the SD rats. No significant differences in the retinal thicknesses were seen between the SDT fatty rats and SDT rats. At 24 weeks, the mean retinal thicknesses 500 microns from the optic nerve disc in the SDT fatty rats, SDT rats, and SD rats, respectively, were 244.5 ± 6.7, 239.3 ± 17.5, and 165.0 ± 3.5 microns (SDT fatty rats vs. SDT rats, *p* = 0.90; SDT fatty rats vs. SD rats, *p* < 0.05; and SDT rats vs. SD rats, *p* < 0.05 by Scheffe's test; SDT fatty rats vs. SDT rats, *p* = 0.52 by Mann–Whitney *U*-test). The choroids tended to be thicker in the SDT fatty rats than in the SD rats. The choroidal thicknesses did not differ significantly between the SDT fatty rats and SDT rats, except for the choroidal thicknesses 500 microns from the optic nerve disc at 24 weeks and choroidal thicknesses 1,000 microns from the optic nerve disc at 16 weeks. At 24 weeks, choroidal thicknesses 500 microns from the optic nerve disc in the SDT fatty rats, SDT rats, and SD rats, respectively, were 13.8 ± 0.2, 11.8 ± 0.6, and 5.9 ± 0.5 microns (SDT fatty rats vs. SDT rats, *p* = 0.19; SDT fatty rats vs. SD rats, *p* < 0.01; and SDT rats vs. SD rats, *p* = 0.16 by Scheffe's test; SDT fatty rats vs. SDT rats, *p* < 0.05 by Mann–Whitney *U*-test).

**Figure 7:**
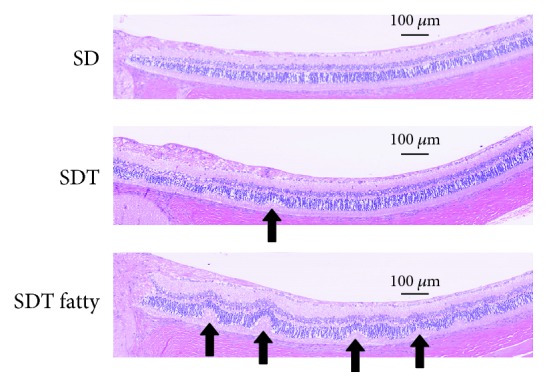
Comparison of the retinal folds in the study animals at 40 weeks of age. The numbers of retinal folds, defined as deformations from the outer nuclear layer to the photoreceptor layer, were measured within 1,500 microns of the optic nerve disc. The retinal folds (arrows) in the Spontaneously Diabetic Torii (SDT) fatty rats are more severe than in the SDT rats. The scale bar indicates 100 microns.

**Figure 8:**
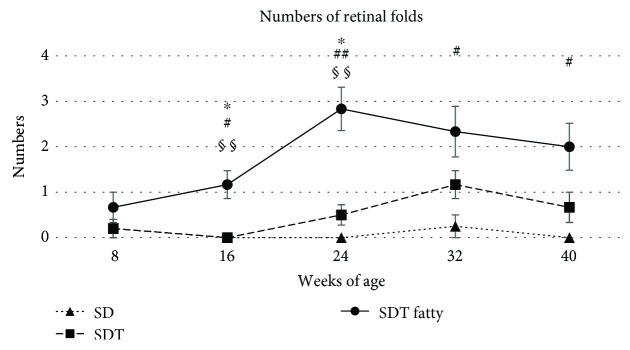
The numbers of retinal folds in the study animals. No retinal folds are seen in the Sprague Dawley (SD) rats. The retinal folds in the Spontaneously Diabetic Torii (SDT) fatty rats developed earlier and are more severe than those in the SDT rats. The data are expressed as the mean ± standard error. ^∗^*p* < 0.05, SDT fatty rats vs. SDT rats; ^#^*p* < 0.05 and ^##^*p* < 0.01, SDT fatty rats vs. SD rats by Scheffe's test. ^§§^*p* < 0.01, SDT fatty rats vs. SDT rats by Mann–Whitney *U*-test.

Figures 7 and 8 show the retinal folds. The retinal folds in the SDT fatty rats developed earlier and were more severe than those in the SDT rats; no retinal folds developed in the SD rats. At 24 weeks, the mean numbers of retinal folds in the SDT fatty rats, SDT rats, and SD rats, respectively, were 2.8 ± 0.5, 0.5 ± 0.2, and 0 ± 0 (SDT fatty rats vs. SDT rats, *p* < 0.05; SDT fatty rats vs. SD rats, *p* < 0.01; and SDT rats vs. SD rats, *p* = 0.58 by Scheffe's test; SDT fatty rats vs. SDT rats, *p* < 0.01 by Mann–Whitney *U*-test). The peaks of the retinal folds occurred at 32 weeks of age in the SDT rats (1.2 ± 0.3) and at 24 weeks of age in the SDT fatty rats (2.8 ± 0.5) (*p* < 0.05, by Mann–Whitney *U*-test).

### 3.3. Areas of Immunostained GFAP and VEGF

**Figure 9:**
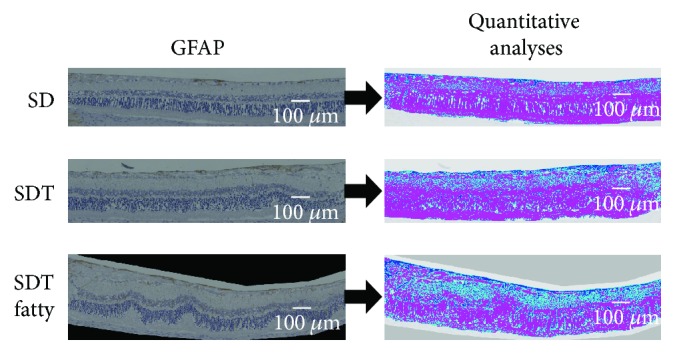
Quantitative analyses of the glial fibrillary acidic protein (GFAP) immunopositive regions were performed within 1,500 microns of the optic nerve disc. The entire specimen is marked with a magenta stain, and the immunopositive regions are marked with dark blue over them. The boundary is marked light blue. The scale bars indicate 100 microns.

**Figure 10:**
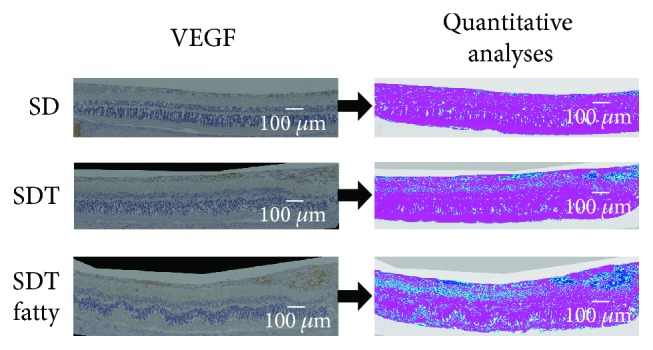
Quantitative analyses of the vascular endothelial growth factor (VEGF) immunopositive regions were performed within 1,500 microns of the optic nerve disc. The entire specimen is marked with a magenta stain, and the immunopositive regions are marked with dark blue over them. The boundary is marked light blue. The scale bars indicate 100 microns.

**Figure 11:**
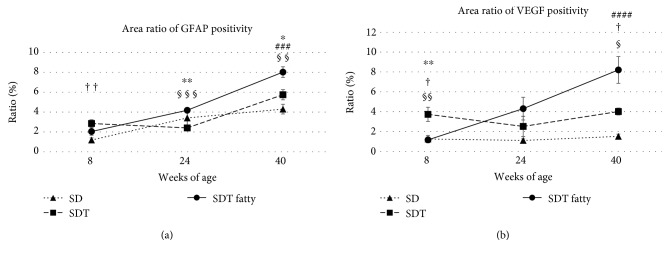
The mean area ratios of glial fibrillary acidic protein (GFAP) and vascular endothelial growth factor (VEGF) positivity ((a) GFAP; (b) VEGF) in the three rat types. Quantitative analysis shows that the GFAP and VEGF immunopositive regions in the retinas of Spontaneously Diabetic Torii (SDT) fatty rats are significantly larger than those of the SDT rats. The data are expressed as the mean ± standard error. ^∗^*p* < 0.05 and ^∗∗^*p* < 0.01, SDT fatty rats vs. SDT rats; ^###^*p* < 0.001 and ^####^*p* < 0.0001, SDT fatty rats vs. Sprague Dawley (SD) rats; ^†^*p* < 0.05 and ^††^*p* < 0.01, SDT rats vs. SD rats by Scheffe's test. ^§^*p* < 0.05, ^§§^*p* < 0.01, and ^§§§^*p* < 0.001, SDT fatty rats vs. SDT rats by Mann–Whitney *U*-test.

Figures 9 and 10 show the areas of immunostained GFAP and VEGF in the rat models.  Figure 11 shows the mean area ratios of immunostained GFAP and VEGF. Quantitative analysis showed that the GFAP and VEGF immunopositive regions in the retinas of the SDT fatty rats were significantly larger than those of the SDT rats. At 40 weeks, the mean area ratios of GFAP positivity in the specimens from the SDT fatty rats, SDT rats, and SD rats, respectively, were 8.0 ± 0.5%, 5.7 ± 0.5%, and 4.3 ± 0.5% (SDT fatty rats vs. SDT rats, *p* < 0.05; SDT fatty rats vs. SD rats, *p* < 0.001; and SDT rats vs. SD rats, *p* = 0.26 by Scheffe's test; SDT fatty rats vs. SDT rats, *p* < 0.01 by Mann–Whitney *U*-test). At 40 weeks, the mean area ratios of VEGF positivity in the SDT fatty rats, SDT rats, and SD rats, respectively, were 8.2 ± 1.4%, 4.0 ± 0.4%, and 1.5 ± 0.2% (SDT fatty rats vs. SDT rats, *p* = 0.29; SDT fatty rats vs. SD rats, *p* < 0.0001; and SDT rats vs. SD rats, *p* < 0.05 by Scheffe's test; SDT fatty rats vs. SDT rats, *p* < 0.05 by Mann–Whitney *U*-test).

## 4. Discussion

In patients with diabetes, longstanding hyperglycemia causes retinal and choroidal thickening because of leakage of the blood components from the retinal and choroidal vessels. Thickened retinas and choroids are seen frequently in clinical observations using OCT. DME, a component of DR, causes visual loss, and this condition is the target of anti-VEGF therapy. We reported previously that the retina and choroid were thicker in the SDT rats compared to the normal nondiabetic SD rats [16, 17]. The choroidal thickening in diabetic eyes remains controversial. Patients with DR have many complications such as hypertension and have been treated with many kinds of treatment such as laser photocoagulation and hypertensive medications. Multiple factors should affect the choroidal vasculature in clinical cases of DR [21–24]. In this particular animal model, the animals had not received any treatment such as laser photocoagulation or medication. Therefore, the animal model has its value. In the current study, the retina and choroid were thicker in the SDT fatty rats than in the SD rats (normal controls). The retinal and choroidal thicknesses did not differ significantly between the SDT fatty rats and SDT rats (controls). However, the numbers of retinal folds and quantitative analysis of the immunohistochemistry showed the progression of DR in SDT fatty rats compared with SDT rats.

The retinal folds in the SDT fatty rats developed earlier and were more severe than those in the SDT rats. In the current study, the retinal folds were defined as deformations observed from the ONL to the PL, changes that did not include the entire retinal layer. Fibrous proliferations and tractional changes were reported at 70 weeks of age in SDT rats, and these changes included the entire retinal layer [11]. These changes usually are seen in older SDT rats; the rats in the current study were too young to have tractional changes. No preretinal membranes, which frequently cause retinal folds in clinical cases, were found in the rats in the current study; the retinal folds did not develop as the result of tractional force but might have resulted from volume changes with edema and/or increasing proliferation in the retina. It has been reported that retinal folds in SDT fatty rats were prevented with phlorizin and ipragliflozin, sodium glucose cotransporter inhibitors [25, 26]. Therefore, the retinal folds would be a phenomenon associated with DR. As mentioned, VEGF and glial cell proliferation should play an important role in retinal edema and retinal folds in both the SDT rats and SDT fatty rats. These mechanisms should have affected the SDT fatty rats more than the SDT rats. We think that retinal folds might be a new indicator for the quantitative assessment of DR in both SDT rats and SDT fatty rats.

Using ImageJ software (National Institutes of Health, Bethesda, MD, USA), we reported that the areas of GFAP and VEGF immunopositivity in the retina were larger in the SDT rats than in the SD rats [17]. In the current study, quantitative analysis of the immunohistochemistry using the Hybrid Cell Count Module/BZ-H3C software showed that the immunopositive regions of the GFAP and VEGF in the retinas were significantly larger in the SDT fatty rats than in the SDT rats, indicating that the SDT fatty rats develop more severe DR earlier than the SDT rats.

To promote drug development, repositioning, and treatments for DR, it is important to objectively evaluate the progress of DR using an appropriate animal model. However, there are few quantitative evaluation methods and few animal models that mimic human DR, which may be responsible for delayed DR research compared to diabetic nephropathy and neuropathy. We quantitatively analyzed the pathological features of DR in SDT fatty rats by measuring the retinal thicknesses, retinal folds, and area ratios of the immunopositive regions. These seem to be useful to objectively evaluate DR. SDT fatty rats are expected to be most useful as a type 2 diabetes animal model to study DR.

For now, the DR found in this SDT fatty rat is the best indicator to represent DR, including DME.

## Data Availability

The data used to support the findings of this study are included within the article.
